# Early Dengue Virus Protein Synthesis Induces Extensive Rearrangement of the Endoplasmic Reticulum Independent of the UPR and SREBP-2 Pathway

**DOI:** 10.1371/journal.pone.0038202

**Published:** 2012-06-04

**Authors:** José Peña, Eva Harris

**Affiliations:** 1 Division of Infectious Diseases and Vaccinology, School of Public Health, University of California, Berkeley, California, United States of America; 2 Graduate Group in Microbiology, Department of Plant and Microbial Biology, University of California, Berkeley, California, United States of America; University of Hong Kong, Hong Kong

## Abstract

The rearrangement of intracellular membranes has been long reported to be a common feature in diseased cells. In this study, we used dengue virus (DENV) to study the role of the unfolded protein response (UPR) and sterol-regulatory-element-binding-protein-2 (SREBP-2) pathway in the rearrangement and expansion of the endoplasmic reticulum (ER) early after infection. Using laser scanning confocal and differential interference contrast microscopy, we demonstrate that rearrangement and expansion of the ER occurs early after DENV-2 infection. Through the use of mouse embryonic fibroblast cells deficient in XBP1 and ATF6, we show that ER rearrangement early after DENV infection is independent of the UPR. We then demonstrate that enlargement of the ER is independent of the SREBP-2 activation and upregulation of 3-hydroxy-3-methylglutaryl-Coenzyme-A reductase, the rate-limiting enzyme in the cholesterol biosynthesis pathway. We further show that this ER rearrangement is not inhibited by the treatment of DENV-infected cells with the cholesterol-inhibiting drug lovastatin. Using the transcription inhibitor actinomycin D and the translation elongation inhibitor cycloheximide, we show that *de novo* viral protein synthesis but not host transcription is necessary for expansion and rearrangement of the ER. Lastly, we demonstrate that viral infection induces the reabsorption of lipid droplets into the ER. Together, these results demonstrate that modulation of intracellular membrane architecture of the cell early after DENV-2 infection is driven by viral protein expression and does not require the induction of the UPR and SREBP-2 pathways. This work paves the way for further study of virally-induced membrane rearrangements and formation of cubic membranes.

## Introduction

Dengue virus (DENV) causes tens of millions of cases of dengue annually and is a major international health concern. DENV is a small, enveloped, positive-sense RNA virus that encodes ten viral proteins – three structural proteins (C, prM/M and E) and seven nonstructural (NS) proteins (NS1, NS2A, NS2B, NS3, NS4A, NS4B and NS5). DENV and other members of the *Flaviviridae* family are endoplasmic reticulum (ER)-tropic viruses that are dependent on the host ER to translate, replicate and package their genome [1]. Infection with DENV, as well as other members of the *Flaviviridae* family, has been shown to induce rearrangement of cellular membranes known as cubic membranes (often described as convoluted membranes and vesicle packets that contain the replication complex) [2], [3], [4]. Recent studies of DENV-2 [5] and WNV_Kun_ [6] using electron tomography revealed the three-dimensional composition of these virally-induced structures to be a network of ER-derived membranes.

The ER is an extensive membranous network that is contiguous with the nuclear membrane and is responsible for the synthesis, maturation and proper folding of a wide range of proteins. It also plays a critical role in Ca^2+^ and lipid homeostasis as well as intracellular signal transduction. When the capacity of the ER to handle client proteins entering the ER is exceeded, this leads to the induction of inter-organelle signaling pathways that respond to ER stress by coordinately regulating translation and gene expression through an elaborate adaptive response known as the unfolded protein response [7]. The unfolded protein response (UPR) functions through the PKR-like ER kinase (PERK), kinase/endoribonuclease inositol requiring 1 (IRE1)/X-box binding protein 1 (XBP1), and/or activation transcription factor 6 (ATF6) pathways. Activation of the UPR slows the flow of nascent proteins entering the ER and allows for the coordinated upregulation of genes involved in maintaining ER homeostasis [7], [8], [9], [10]. Activation of transcription factors XBP1 and ATF6 has been recently linked to ER biogenesis, indicating that the UPR can also expand and increase the size of the ER [11], [12], [13], [14]. Despite the UPR’s protective role, prolonged and persistent ER stress can result in caspase activation and cellular apoptosis [15].

Additionally, the ER houses the sterol regulatory element-binding protein (SREBP) sensors that are important for maintaining cholesterol and lipid metabolism through the SREBP pathway [16]. SREBPs are ER membrane-associated transcription factors and include SREBP-1 and SREBP-2. SREBP-1 consists of two isoforms, SREBP-1a and SREBP-1c, due to alternative splicing. SREBP-1 is involved in the biosynthesis of cholesterol and fatty acids, while SREBP-2 primarily mediates cholesterol biosynthesis [17]. SREBP proteins are maintained in an inactive state via association with SREBP-cleavage-activating protein (Scap), which functions as a sterol sensor, and another ER-associated protein, insulin-induced gene (Insig). When ER membranes are depleted of cholesterol, Scap escorts SREBP from the ER to the Golgi, where it is proteolytically processed and activated [16], [18], [19], [20]. Transcriptionally active SREBPs also enhance their own transcription through a feed-forward mechanism that is dependent on the amount of cholesterol present in the cell [21].

The rearrangement and formation of virally-induced membrane structures have been reported for DENV and various positive-strand RNA viruses [2], [3], [4], [22], [23], [24], [25]. However, despite our improved understanding of the composition and three dimensional architecture of these structures, how they are formed, the requirements for their formation, the involvement of viral and host factors, and the induction of cellular pathways involved in lipid and ER biogenesis are still not well defined. In these studies, we investigate which cellular pathways are involved in the rearrangement and expansion of the ER early after DENV-2 infection by examining cellular pathways previously reported to induce lipid and ER membrane biogenesis. Recently, we reported the time-dependent activation of the UPR during a DENV-2 infection [26]. Here, through the use of high-resolution laser scanning confocal microscopy and differential interference microscopy, we show that DENV induces rearrangement and expansion of the ER early after infection independently of the UPR or SREBP2 pathways. Rather, the gross enlargement of the ER early in DENV-2 infection appears to be driven by the expression of viral proteins, causing the rearrangement of the preexisting ER membranous network and the reabsorption of lipid droplets (LDs).

## Results

### Dengue Virus Induces ER Expansion Early After Infection

**Figure 1:**
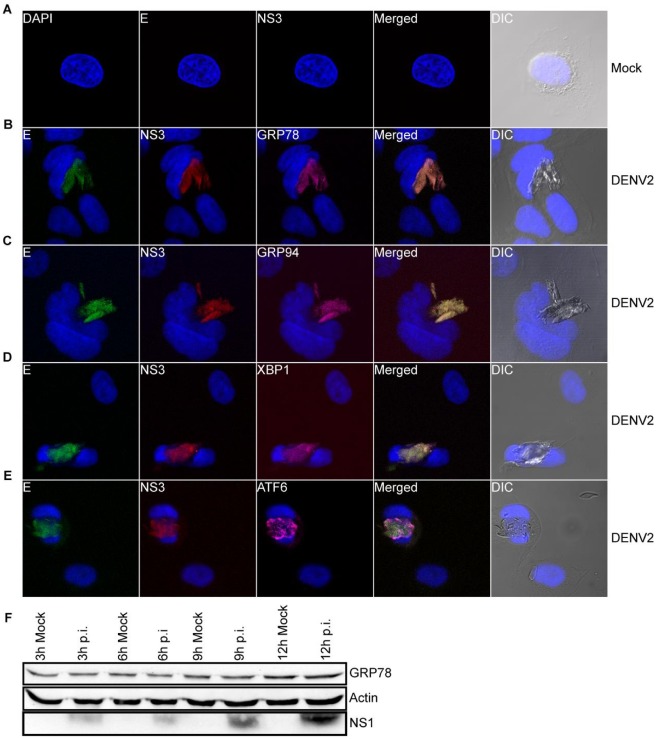
DENV induces rearrangement of the ER early after infection. 2fTGH cells were (A) mock-infected or (B–E) infected with DENV-2 for 12 h and stained intracellularly for UPR markers and viral proteins E and NS3. (A) Mock-infected 2fTGH cells were stained with mouse MAbs against DENV E (4G2; green) and NS3 (E1D8; red) directly conjugated to Alexa Fluor® 488 and Alexa Fluor® 594, respectively. Nuclear staining (blue) was performed using DAPI stain. Intracellular staining of DENV-infected cells was carried out as in (A); in addition primary antibodies specific for cellular proteins (B) GRP78, (C) GRP94, (D) XBP1 and (E) ATF6 (magenta) were used, followed by secondary antibodies conjugated to Alexa Fluor® 647. The multiple-exposure images were captured using 63X/1.25 Plan-Neofluar DIC oil objective on Zeiss 510 LSM using bandpass filter sets appropriate for DAPI, Alexa Fluor® 488, Alexa Fluor® 594 and Alexa Fluor® 647. For individual images, the additional channels were turned OFF post-acquisition using the Zeiss operating software. Co-localization is observed in the “Merged” image as a yellow hue. Merged and DIC images represent a single image with all detector channels ON. Total magnification 630×. (F) Western blot analysis of GRP78 over a 12-h course of DENV infection. Actin was used as a loading control, and expression of DENV NS1 was used to confirm viral infection.

We began investigating ER expansion early after DENV-2 infection by testing two hypotheses. The first posits that the rearrangement and expansion of the ER leads to the concentration of resident ER proteins within the enlarged ER without inducing increased transcription and/or translation of resident ER proteins or additional membrane biogenesis. Alternatively, the second hypothesis is that the rearrangement and expansion of the ER early during DENV-2 infection requires the induction of pathways involved in lipid and ER biogenesis. To evaluate the role of the UPR in ER expansion early after DENV-2 infection, we first stained cellular proteins ATF6 and XBP1 in uninfected human 2fTGH cells that were mock-treated (Figure S1 A) or treated with DTT (Figure S1 B), a potent ER stress inducer. Consistent with our previous results, we found that UPR induction using DTT leads to the activation and nuclear localization of XBP1 and ATF6 (Figure S1 B) [26]. Next, we mock-treated or treated uninfected 2fTGH cells with the ER stress-inducing drug thapsigargin for 12 hours (h) to mimic persistent ER stress and trigger the upregulation of the resident ER chaperone proteins GRP78 and GRP94. Under these conditions, we demonstrate that GRP78 (Figure S1 C) is strongly induced; however, induction of GRP94 is minimal (Figure S1 D), compared to mock-treated cells (Fig. 1E and 1F; respectively). As a negative control for viral infection, we also stained uninfected and mock-treated 2fTGH cells for DENV E and NS3, and as expected, no staining was observed (Fig. 1A and Figure S1 C and D).

Having shown the specificity and cellular distribution of these UPR markers, we proceeded to test our two hypotheses. We previously demonstrated the induction of the IRE1-XBP1 pathway 12–36 h post-infection (p.i) and the transient activation of the ATF6 pathway late in DENV-2 infection [26]. Here, we focused on a very early time-point, 12 h p.i. Through the use of laser scanning confocal microscopy (LSCM) and differential interference contrast (DIC) microscopy, we show the ER-resident chaperone proteins GRP78 (Fig. 1B) and GRP94 (Fig. 1C) to be localized within an enlarged ER in DENV-2-infected 2fTGH cells at 12 h p.i. These ER-resident chaperone proteins colocalize with DENV-2 viral proteins E and NS3 within a grossly enlarged ER that is visible using DIC imaging (Fig. 1B, C, D, E). This gross alteration of the ER is absent in mock-infected 2fTGH cells (Fig. 1A), neighboring uninfected cells (Fig. 1B and 1C), and thapsigargin (Tg)-treated cells (Figure S1 C and D).

In our previous studies, we demonstrated that *Xbp1* mRNA splicing began 12 h after DENV-2 infection [26]. Based on these observations, we next wanted to determine whether the activation of the IRE1-XBP1 pathway was driving the rearrangement and expansion of the ER at this early time-point during a DENV-2 infection. When we stained DENV-2-infected 2fTGH cells for XBP1, we found XBP1 to be expressed; however, it co-localized with NS3 and E within the enlarged ER (Fig. 1D) and not in the nucleus, as demonstrated in our DTT activation control (Figure S1 B). Additionally, we wanted to show that the ATF6 pathway was not induced at 12 h p.i. Using an N-terminus-specific anti-ATF6 antibody that recognizes the cytosolic portion of ATF6, we demonstrated that activation of the ATF6 pathway is not induced, as it remained localized to the ER and did not translocate to the nucleus (Fig. 1E), again consistent with our previous results [26].

The observation that both ER-resident proteins GRP78 and GRP94 in DENV-2-infected 2fTGH were colocalized to the ER with viral proteins E and NS3 in the absence of XBP1 or ATF6 nuclear localization suggested that ER rearrangement and expansion during DENV-2 infection proceeded independently of the UPR. However, it has been demonstrated that GRP78 contains an IRES element that allows for increased translation without inducing transcriptional activation [27], [28]; therefore, we investigated whether GRP78 expression was upregulated early during a DENV-2 infection. When we infected 2fTGH cells with DENV-2 at an MOI of 5 for a 3–12 h time-course and analyzed the lysates by immunoblot for levels of GRP78, we found no difference in protein levels in DENV-2-infected cells compared to mock-infected cells (Fig. 1F); this observation is also consistent with our previous findings [26]. These results suggest that DENV leads to the rearrangement and expansion of the ER early after infection and that the fluorescence increase we observed in the ER results from the relocalization and concentration of GRP78 and GRP94 within the expanded ER without *de novo* protein synthesis.

### DENV-2 Induces ER Expansion in a UPR-independent Manner Early in Infection

**Figure 2:**
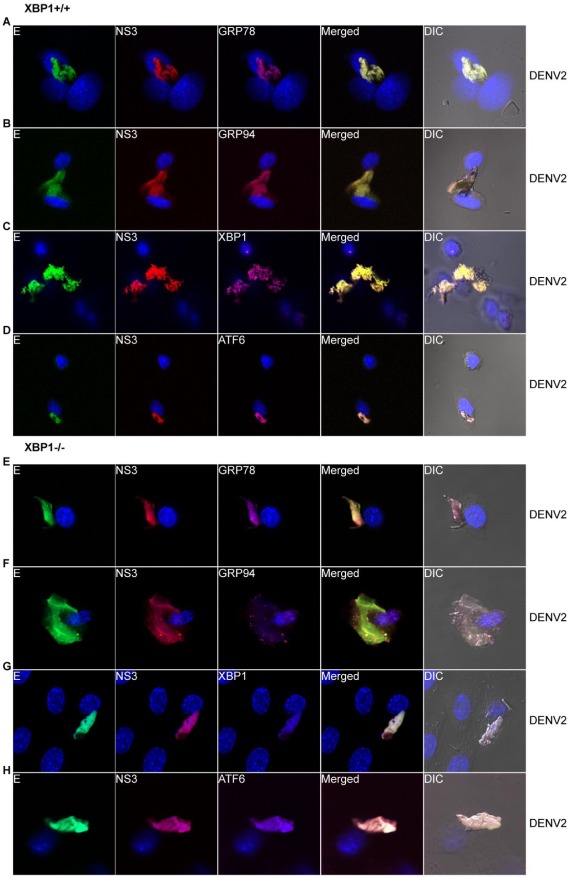
DENV-induced ER rearrangement and expansion is independent of the XBP-1 pathway. XBP1^+/+^ (A–D) and XBP1^−/−^ (E–H) MEF cells were infected with DENV-2 for 12 h and stained intracellularly for cellular proteins using antibodies against (A and E) GRP78, (B and F) GRP94, (C) XBP1, and (G) ATF6 (magenta), followed by secondary antibodies conjugated to Alexa Fluor® 647. DENV proteins were detected with mouse MAbs against DENV E (green) and NS3 (red) directly conjugated to Alexa Fluor® 488 and Alexa Fluor® 594, respectively. Nuclear staining (blue) was performed using DAPI stain. Images were acquired and processed as described in Fig. 1. Total magnification 630×.

Previous studies have shown XBP1 to play a critical role in cell size expansion, organelle biogenesis, increased protein synthesis, and induction of secretory genes [11], [13], [14], [29]. In Fig. 1, we presented data that suggests that DENV-induced rearrangement and expansion of the ER is independent of the UPR. To further demonstrate that this ER rearrangement is independent of the XBP1 pathway, we infected XBP1^+/+^ and XBP1^−/−^ mouse embryo fibroblast (MEF) cells with DENV-2 for 12 h and fixed and processed them for LSCM analysis. As demonstrated with 2fTGH cells (Fig. 1), we found that ER-resident proteins GRP78 and GRP94 co-localize with E and NS3 proteins within the enlarged ER of DENV-2-infected XBP1^+/+^ (Fig. 2A and 2B) and XBP1^−/−^ (Fig. 2E and 2F) MEF cells. As expected, XBP1 is visible in DENV-2-infected XBP1^+/+^ MEF cells (Fig. 2C) where we again find it colocalized with E and NS3 in the ER, but it is not detected in XBP1^−/−^ MEF cells (Fig. 2G). A recent study demonstrated that the ATF6 pathway can induce expansion and upregulation of pathways involved in lipid biosynthesis and ER biogenesis in XBP1^−/−^ MEFs [12]. When we examined the activation of the ATF6 pathway in both XBP1^+/+^ (Fig. 2D) and XBP1^−/−^ (Fig. 2H) MEF cells, we found that ATF6 remains uninduced and localized to the ER of DENV-2-infected cells, also consistent we our previous results [26].

**Figure 3:**
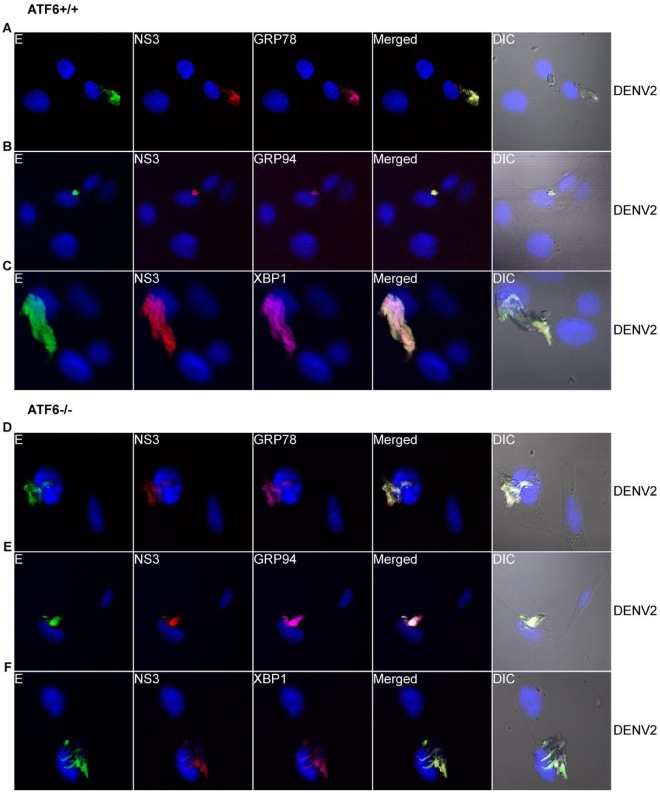
DENV-induced ER rearrangement and expansion is independent of the ATF6 pathway. ATF6^+/+^ (A–C) and ATF6^−/−^ (D–F) MEF cells were infected with DENV-2 for 12 h and intracellularly stained for cellular proteins (A and D) GRP78, (B and E) GRP94, and (C and F) XBP1 (magenta), followed by secondary antibodies conjugated to Alexa Fluor® 647. DENV proteins were detected with mouse MAbs against DENV E (green) and NS3 (red) directly conjugated to Alexa Fluor® 488 and Alexa Fluor® 594, respectively. Nuclear staining (blue) was performed using DAPI stain. Images were acquired and processed as described in Fig. 1. Total Magnification 630×.

To further demonstrate that rearrangement and expansion of the ER is ATF6-independent early after DENV-2 infection, we infected ATF6^+/+^ and ATF6^−/−^ MEF cells with DENV-2, fixed them, and processed them for LSCM 12 h p.i. Consistent with the data in Fig. 1, we once again found expression of GRP78 (Fig. 3A and 3D) and GRP94 (Fig. 3B and 3E) to localize within a rearranged and enlarged ER in DENV-2-infected ATF6^+/+^ and ATF6^−/−^ MEF cells. Furthermore, expression of DENV E and NS3 proteins was co-localized with GRP78 and GRP94 in the expanded ER. In addition, the expression of XBP1 is induced but remains localized to the ER in both ATF6^+/+^ (Fig. 3C) and ATF6^−/−^ (Fig. 3F) MEF cells. These data show that rearrangement and expansion of the ER early during DENV-2 infection is independent of the ATF6 and IRE1/XBP1 pathways of the UPR.

### DENV-2 Induces ER Expansion in an SREBP-2-independent Manner

**Figure 4:**
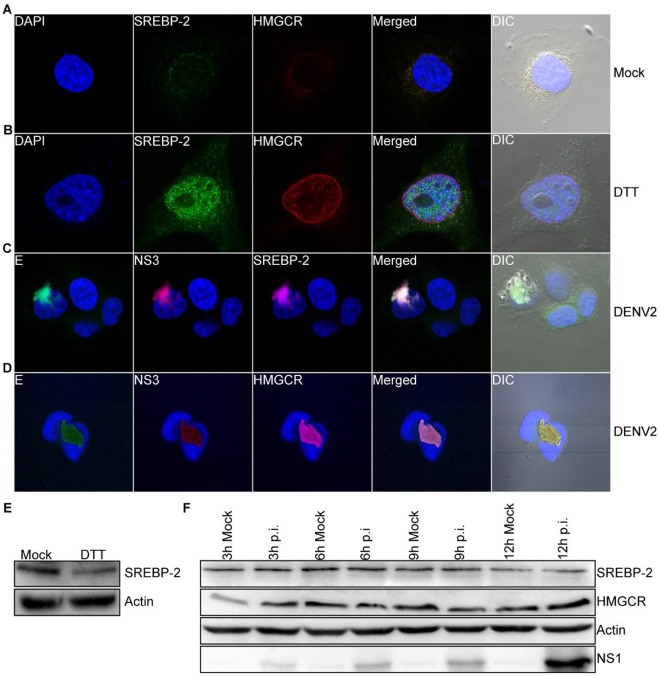
DENV does not induce the SREBP-2 pathway or upreglation of HMGCR early after infection. 2fTGH cells were mock-treated (A) or treated with DTT (B) for 6 h. Cells were then fixed and stained intracellularly for cellular proteins using antibodies against SREBP-2 (green) and HMGCR (red) followed by secondary antibodies conjugated to Alexa Fluor® 488 and Alexa Fluor® 546, respectively. 2fTGH cells were infected with DENV-2 for 12 h and stained intracellularly for (C) SREBP-2 (magenta) and (D) HMGCR (magenta) followed by secondary antibodies conjugated to Alexa Fluor® 647; mouse MAbs against DENV E (green) and NS3 (red) directly conjugated to Alexa Fluor® 488 and Alexa Fluor® 594, respectively, were used to detect DENV proteins. Nuclear staining (blue) was performed using DAPI stain. The multiple-exposure images were captured using a 40×/1.4 Plan-Apochromat DIC oil objective on a Zeiss 710 LSM using bandpass filter sets appropriate for DAPI, Alexa Fluor® 488, Alexa Fluor® 594 and Alexa Fluor® 647 and were processed as previously described in Figure 1. (E) SREBP-2 proteolyic processing by immunoblot analysis. Actin was used as a loading control. In (F), 2fTGH cells were infected with DENV-2 over a 3–12 h time-course. Lysates were collected and analyzed for SREBP-2 and HMGCR expression by immunoblot analysis. Actin was used as a loading control, and expression of DENV NS1 was used to confirm viral infection. A time-matched mock-infected control was included at each time-point. Total magnification 600×.

SREBP-2 has been shown to regulate both lipid and cholesterol synthesis *in vivo* and *in vitro* [30], [31], [32]. Therefore, we next investigated whether DENV-2 infection led to activation of the SREBP-2 pathway and induction of 3-hydroxy-3-methylglutaryl-Coenzyme A reductase (HMGCR), the rate-limiting enzyme involved in cholesterol biosynthesis. We first mock-treated or DTT-treated 2fTGH cells for 6 h to induce ER stress and stained for cellular distribution of SREBP-2 and HMGCR. Mock-treated 2fTGH cells demonstrated diffused staining of SREBP-2 throughout the cell, while low levels of HMGCR were detected around the nucleus (Fig. 4A). This contrasts with DTT-treated 2fTGH cells (Fig. 4B), which demonstrated activation of SREBP-2 by increased nuclear localization and increased expression of HMGCR around the nucleus. We next determined whether rearrangement of the ER during DENV-2 infection was driven by the activation of SREBP-2 and upregulation of HMGCR in infected cells. We infected 2fTGH cells with DENV-2 at an MOI of 5 for 12 h, fixed and stained the cells, and determined whether the SREBP-2 pathway was activated and expression of HMGCR upregulated. SREBP-2 staining of DENV-2-infected 2fTGH cells (Fig. 4C) demonstrated that SREBP-2, like ATF6, remained inactive and localized to the ER with E and NS3 viral proteins. HMGCR was redistributed to the enlarged ER in DENV-2-infected cells compared to its nuclear distribution in uninfected cells (Fig. 4D).

To further demonstrate that early DENV-2 infection did not lead to the activation of the SREBP-2 pathway, we mock-treated or DTT-treated 2fTGH cells for 6 h, harvested lysates, and processed them by immunoblot to monitor proteolytic processing of the precursor SREBP-2 (pSREBP-2). As shown in Figure 4E, cells that were treated with DTT displayed lower levels of pSREBP-2 than mock-treated cells, consistent with previously published results [33]. We next mock-infected these cells or infected them with DENV-2 and carried out a 3–12 h course of infection. Cells were harvested at the indicated time-points, and lysates were processed for immunoblot. As shown in Figure 4F, levels of pSREBP-2 in DENV-2-infected cells were not reduced compared to the time-matched mock-infected controls, and levels of HMGCR remained essentially unchanged. To demonstrate that cells were infected, we probed for viral NS1 protein expression. Together, these data show that rearrangement and expansion of the ER early during DENV-2 infection proceeds independently of SREBP-2 activation and induction of HMGCR. Previous studies have demonstrated HMGCR to be synthesized and localized to the outer nuclear membrane [34]. Our data demonstrate the cellular redistribution of HMGCR to the ER with E and NS3 viral proteins, consistent with previous results for WNV_kun_ [35] and suggest a potential involvement of the outer nuclear membrane in the enlargement and expansion of the ER during DENV infection.

### Lovastatin Inhibits Production of Infectious Virus but not ER Rearrangement and Expansion

**Figure 5:**
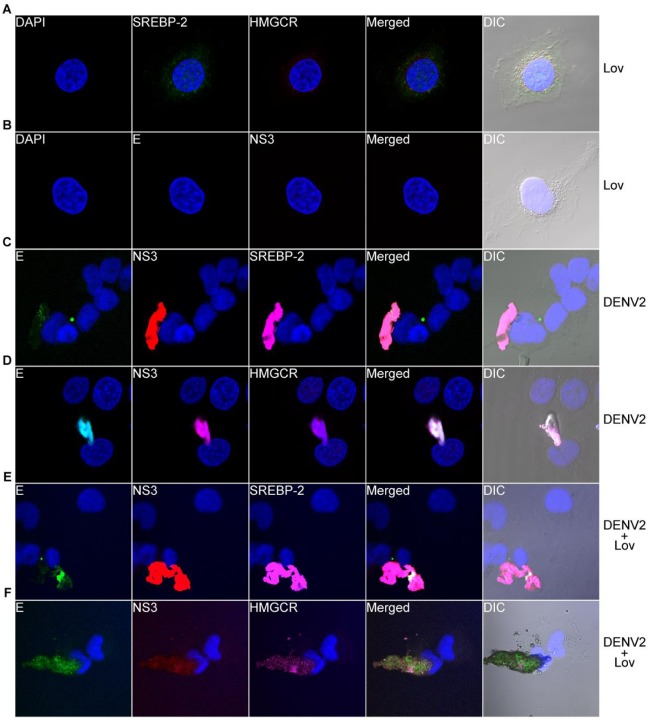
Lovastatin treatment of DENV-infected cells does not inhibit ER rearrangement. Mock-infected 2fTGH cells were treated with 10 µM lovastatin for 12 h and were stained intracellularly for (A) SREBP-2 (green) and HMGCR (red). (B) Cells were also stained for DENV proteins E (green) and NS3 (red). 2fTGH cells were infected with DENV-2 and mock-treated for 12 h. Cells were fixed and stained intracellularly for (C) SREBP-2 (magenta) and (D) HMGCR (magenta), followed by DENV E (green) and NS3 (red) staining. Nuclear staining (blue) was performed using DAPI. DENV-infected 2fTGH cells were treated with 10 µM lovastatin at the time of infection and incubated for 12 h. Cells were fixed and stained as described above for (E) SREBP2 (magenta) and (F) HMGCR (magenta) and DENV E (green) and NS3 (red). Nuclear staining (blue) was performed using DAPI, and images were acquired as previously described. Total magnification 630×.

Our studies thus far have demonstrated that DENV-2 infection leads to early rearrangement and expansion of the ER that is independent of the UPR and SREBP-2 pathways. However, recent studies on WNV_kun_, JEV, and DENV have shown cholesterol to play an important role in viral entry, replication and immune evasion [35], [36], [37], [38]. Thus, we next evaluated whether treatment of 2fTGH cells with lovastatin, a cholesterol inhibitor, prevented the rearrangement of the ER in cells infected with DENV-2. To test this, we first mock-treated or lovastatin-treated 2fTGH cells for 12 h. Cells were fixed and stained for SREBP-2 and HMGCR and analyzed by LSCM and DIC. Treatment of mock-infected 2fTGH cells with 10 µM lovastatin did not lead to activation of SREBP-2 or upregulation of HMGCR (Fig. 5A), nor to the rearrangement and expansion of the ER. Staining for DENV E and NS3 was negative, as expected (Fig. 5B). We then tested whether lovastatin inhibited DENV-2-induced ER expansion. 2fTGH cells were mock-infected or infected with DENV-2 at an MOI of 5 for 12 h in the presence or absence of 10 µM lovastatin at the time of infection. Cells were fixed and stained for SREBP-2 and HMGCR as well as for viral proteins E and NS3. As with our previous results, we demonstrated that DENV-2 infection leads to the rearrangement and expansion of the ER (Fig. 5C and 5D). Also, SREBP-2 (Fig. 5C) and HMGCR (Fig. 5D) co-localized to the enlarged ER with E and NS3 as previously shown in Fig. 4. The treatment of DENV-2-infected 2fTGH cells with 10 µM lovastatin at the time of DENV-2 infection did not lead to the activation of the SREBP-2 pathway (Fig. 5E) or upregulation of HMGCR (Fig. 5F). Moreover, the treatment of DENV-2-infected cells with lovastatin did not inhibit the rearrangement and expansion of the ER (Fig. 5E and 5F).

The observation that lovastatin did not inhibit DENV-induced ER rearrangement was an interesting finding given that previous studies have demonstrated lovastatin to inhibit infectious virus production. We next tested whether lovastatin inhibited DENV-2 infectious virus production in our 2fTGH system [38]. We carried out a 24–48 h course of infection in which 2fTGH cells were infected with DENV-2 at an MOI of 5 in the presence or absence of increasing concentrations of lovastatin. As demonstrated in Figure S2, production of infectious virus decreased in a dose-dependent manner, with 10 µM lovastatin achieving the highest inhibition of viral replication at both 24 and 48 h p.i. These results are consistent with those published for DENV-2 strain New Guinea C [38].

### DENV-2 Protein Synthesis is Necessary to Induce ER Rearrangement and Expansion

**Figure 6:**
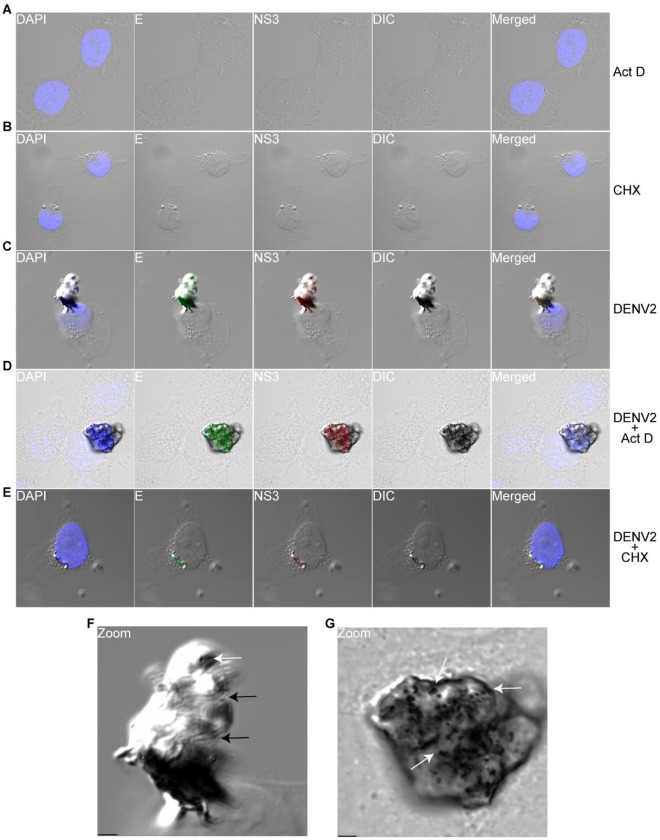
DENV-induced ER rearrangement requires viral protein synthesis. Mock-infected 2fTGH cells were (A) treated with 10 µg/mL actinomycin D (Act D) or (B) treated with 50 µg/mL cycloheximide (CHX), fixed and stained intracellularly for viral proteins E (green) and NS3 (red); nuclear staining (blue) was performed with DAPI. DENV-infected 2fTGH cells were (C) mock-treated or treated with (D) Act D or (E) CHX at the time of infection. Cells were fixed and stained as in A and B. Figures (F) and (G) are increased magnification of figures C and D, respectively. Black arrows point to lamellar stack-like structures and white arrows point to vesicle-like pore openings on the enlarged ER. Scale bar  = 2 µm. Total magnification 600×.

We next wondered whether other cellular pathways might be involved in the rearrangement and expansion of the ER in DENV-2 infected cells or whether viral protein synthesis was sufficient to drive this ER alteration. We tested these possibilities by examining whether inhibition of cellular transcription or viral protein synthesis would abrogate the rearrangement and expansion of the ER in cells infected with DENV-2. To do this, we first tested whether treatment of 2fTGH cells with actinomycin D (Act D) would inhibit the upregulation of GRP78 in the presence of the ER stress-inducing drug thapsigargin (Tg). Treatment of cells with Tg has been shown to upregulate transcription and translation of GRP78 [39]. 2fTGH cells were mock-treated or treated with 1 µM Tg or 10 µg/mL Act D or both for 12 h, and levels of GRP78 were analyzed by immunoblot. As demonstrated in Figure S3, cells treated with Tg demonstrated elevated levels of GRP78 expression compared to mock-treated or Act D-treated cells. In contrast, levels of GRP78 remained unchanged in cells that were co-treated with Tg and Act D. From these results, we concluded that the Act D concentration and duration of treatment were adequate for our experiments. We next mock-infected or DENV2-infected 2fTGH cells for 12 h in the presence or absence of 10 µg/mL Act D or 50 µg/mL cycloheximide (CHX) at the time of infection. Cells were fixed and stained for viral proteins E and NS3. Staining of mock-infected 2fTGH cells that were mock-treated or treated with Act D (Fig. 6A) or CHX (Fig. 6B) demonstrated no E or NS3 staining and there was no gross enlargement of the ER. When we stained for E and NS3 expression in DENV-2 infected cells treated with Act D, we found them to be expressed (Fig. 6D). Moreover, we found that DENV-2 infected cells treated with Act D had a grossly enlarged ER (Fig. 6D) similar to DENV-2 infected cells that were mock-treated (Fig. 6C). In contrast, treatment of DENV-2-infected 2fTGH cells with CHX resulted in the inhibition of viral protein synthesis and in the few infected cells where some viral translation occurred, we observed a drastic reduction in viral protein expression and in the rearrangement and expansion of the ER (Fig. 6E) compared to DENV-2-infected 2fTGH cells that were mock-treated (Fig. 6C) or treated with Act D (Fig. 6D). Increased magnification of DENV-2 infected cells that were mock-treated revealed what appear to be lamellar stacks (Fig. 6F), and vesicle pore-like openings [5] were evident in DENV-2-infected cells that were mock-treated (Fig. 6F) or treated with Act D (Fig. 6G). These data demonstrate that rearrangement and expansion of the ER during DENV-2 infection does not require the *de novo* expression of host genes involved in ER biogenesis. Moreover, it shows that DENV-2 viral protein synthesis is necessary to induce rearrangement of the ER.

### DENV-2 Induced ER Rearrangement and Expansion Induces Lipid Droplet Reabsorption

**Figure 7:**
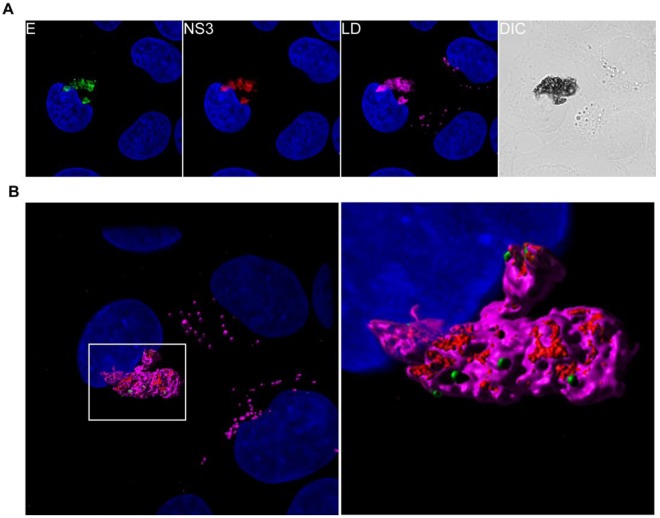
DENV-induced ER expansion induces lipid droplet reabsorption. DENV-2-infected 2fTGH cells were fixed and stained with mouse MAbs against DENV-2 E-protein (4G2; pseudo-colored green) and NS3 (red). Lipid droplets (LD; pseudo-colored magenta) were stained using Bodipy 493/503 dye. (B) Three-dimensional reconstruction of the rearranged and expanded ER early after DENV-2 infection. The deconvolved image was rotated 180° on the X-axis relative to Fig. 7A. Images were obtained using a Zeiss 710 LSM as previously described. Z-stack series (61 panels) were deconvolved using Huygens software (Scientific Volume Imaging), and an iso-surface model was generated using Imaris 7.2 software (Bitplane Scientific Software). LD (pseudo-color magenta), E (pseudo-color green) and NS3 (red). Total magnification 600×.

In this study, we have demonstrated that DENV-2-induced ER rearrangement and expansion proceeds independently of cellular pathways involved in ER biogenesis. Additionally, our data demonstrates that DENV viral protein synthesis is sufficient to drive the rearrangement of preexisting intracellular membranes. However, the data presented so far do not fully account for the increased volume of the ER during DENV-2 infection. Given the sudden cellular demand for lipids after DENV infection and the non-involvement of the UPR and SREBP-2 pathways, we decided to investigate whether lipid droplets (LDs) might provide the cell with a pool of ready-made lipids for the expansion of the ER. LDs are organelles specialized for the storage of neutral lipids and regulation of cellular lipid metabolism, and they function as lipid processing centers that are readily available to the cell [40]. Therefore, we infected 2fTGH cells with DENV-2 for 12 h, fixed them, and stained for viral proteins E and NS3 and for LDs with Bodipy 493/503, a dye that specifically targets neutral lipids in LDs, and processed the cells for LSCM. Uninfected cells showed a staining pattern characteristic of LDs (Fig. 7A). Interestingly, these LD spots were not seen in DENV-infected cells; instead, we found the ER to be stained with the Bodipy 493/503 dye, suggesting the LD neutral lipids had been reabsorbed into the ER (Fig. 7A). As with our previous results, staining of E and NS3 viral proteins colocalized to the grossly enlarged ER of DENV-2 infected cells and was absent in adjacent non-infected cells (Fig. 7A). In order to gain more accurate information regarding the spatial localization of LDs in uninfected cells and the relative rearrangement of the ER in DENV-2-infected cells, we performed a deconvolution of the image stacks from Figure 7A and generated three-dimensional (3-D) views of the deconvoluted data with an iso-surface rendering based on the LDs stained with Bodipy 493/503. This 3-D image rendition allows for the visualization of the LDs in the uninfected cells while also allowing for the visualization of the neutral lipid staining within the enlarged ER as well as the DENV-2 viral proteins in the infected cells. In agreement with Figure 7A, LDs are only found in uninfected cells (Fig. 7B). These results suggest an early involvement of LDs in the rearrangement and expansion of the ER.

## Discussion

The results presented here for DENV-2 support our first hypothesis; namely, that viral protein synthesis is sufficient to drive the early expansion of the ER without inducing increased transcription and/or translation of resident ER proteins and genes involved in membrane biogenesis. Early after infection (12 h), DENV did induce the IRE1-XBP1 pathway of the UPR, but expression of XBP1 was localized to the ER and not the nucleus. These data are consistent with our previous results that demonstrate splicing of the precursor mRNA of *Xbp1* as early as 12 h p.i. [26]. Using genetically-deficient cells, we show that the early DENV-induced ER expansion occurrs independently of the XBP-1 and ATF6 pathways. Additionally, we demonstrate that DENV-2 infection at 12 h p.i. does not induce the SREBP-2 pathway or upregulation of HMGCR. Although the data presented here did not support our second hypothesis that the induction of cellular stress pathways such as the UPR and lipid biosynthesis (e.g., SREBP-2) is necessary for rearrangement and expansion of the ER early after DENV-2 infection, these two hypotheses are not mutually exclusive in that cellular stress pathways do play a role as the infection progresses. This interpretation is consistent with our previous studies, in which we demonstrated that DENV-2 infection modulates the UPR in a time-dependent manner by sequentially activating and suppressing PERK-eIF2α phosphorylation early in infection, followed by the activation of the IRE1-XBP1 pathway during mid-infection and then activation of the ATF6 pathway and SREBP-2 pathway late in infection [26]. The sequential activation of these pathways is further supported by studies with WNV_kun_, which showed that HMGCR is upregulated at later times post-infection [35]. We believe the early rearrangement of the ER provides the virus with the initial membranous platform necessary to establish a productive infection and localizes and concentrates preexisting cellular proteins required for viral translation, replication and immune evasion. Not surprisingly, viruses that target the ER have evolved to take advantage of this organelle to propagate more effectively [41], [42], [43], [44], [45].

Cells infected with a wide range of viruses have been shown to accumulate rearranged and expanded ER and other cellular membranes [25]. For the *Flaviviridae* family of viruses, the rearranged and expanded ER is often referred to as convoluted membranes, paracrystalline arrays, lamellar structures or vesicle packets [25]. However, little is known as to how proteins encoded by members of the *Flaviviridae* family induce rearrangement of the ER. The NS4A protein of WNV_kun_ and DENV-2 [46], [47], as well as Hepatitis C virus (HCV) NS4B [48], have been shown to induce ER rearrangement when overexpressed. DENV NS3 protein has been shown to be associated with the nuclear receptor binding protein and to induce ER membrane alteration and proliferation [49]. One study of HCV demonstrated that overexpression of all viral proteins individually or in combination, with the exception of NS5A and NS5B, led to distinct membrane alterations, indicating that both homotypic and heterotypic protein interactions can modulate membrane rearrangement and expansion [50]. Likewise, overexpression of ER-associated proteins are involved in driving ER rearrangement and expansion [23]. HMGCR, rat microsomal aldehyde dehydrogenase (msALDH), cytochrome P450 and cytochrome b, for example, have been shown to form crystalloid ER, also referred to as organized smooth ER (OSER), when overexpressed [51], [52], [53], [54]. These OSER-forming proteins are anchored to the ER via transmembrane domains, and a large portion of their cytoplasmic domain is exposed. Mutational analysis of both these domains has been shown to abolish OSER formation, thus demonstrating that ER localization and homotypic protein interactions play an important role in rearrangement of the ER [23]. Our data indicate that synthesis and targeting of viral proteins early in infection are sufficient to drive the rearrangement of the ER, presumably via cytoplasmic heterotypic protein interactions. This rearrangement of the ER leads to redistribution of HMGCR from the outer membrane of the nuclear envelope and to the clustering of ER-resident proteins along with viral proteins. The degree of affinity between the cytoplasmic domains of viral and cellular proteins could explain the different shapes and sizes of the ER structures that are observed projected in three-dimensional space during viral infection in this and other studies [3], [4], [22], [23], [25]. We hypothesize that homotypic and heterotypic protein interactions between viral and cellular proteins drive the rearrangement of the ER at this early stage in DENV infection.

Recent studies with DENV, JEV, WNV_kun_ and WNV_NY99_ have implicated cholesterol to play a major role in viral infection. Cholesterol depletion with methyl-β-cyclodextrin (MβCD) resulted in disruption of lipid rafts on the plasma membrane, effectively blocking entry of WNV_NY99_, JEV and DENV-2 [36], [37]. Studies with WNV_kun_ have shown the redistribution of cholesterol from the plasma membrane to membranous sites of viral replication and targeting of proteins involved in cholesterol biosynthesis, namely HMGCR, to these viral replication complexes [35]. However, a different study with WNV_NY99_ did not find the cholesterol biosynthesis pathway to be induced, suggesting that *de novo* cholesterol synthesis might not be needed for WNV RNA replication [37]. In these studies, we find that 12 h p.i, DENV-2-induced ER rearrangement leads to the redistribution of HMGCR from the outer membrane of the nuclear envelope, where it is synthesized and localized [34] to sites of viral replication, in agreement with previous observations [35]. However, we did not observe activation of SREBP-2 or induction of HMGCR expression at this early time-point. Furthermore, when we treated cells with lovastatin to inhibit *de novo* cholesterol biosynthesis, we were still able to detect ER membrane rearrangement and found SREBP-2 to remain inactive but redistributed and colocalized with viral proteins E and NS3. This further suggests that initial intracellular cholesterol levels are sufficient to maintain membrane fluidity and formation of viral replication sites, in agreement with the results of Medigeshi *et al* [37]. Although our studies did not specifically focus on the lipid biosynthesis pathways, our findings that SREBP-2, also a transcription activator of fatty acid synthase (FASN) [55], [56], is not induced at this early time-point is also in agreement with previous studies in which FASN was shown to be redistributed to sites of viral replication but was not upregulated during DENV infection [57]. Additionally, our data show that although treatment with lovastatin does lower infectious viral production at later time-points, as previously reported [38], rearrangement and expansion of the ER are still induced in the presence of this drug, implying that cholesterol is important for viral production, such as assembly or egress, but not at the step of inducing ER rearrangement early in DENV-2 infection.

The central of role of LDs has become the focus of many studies, from the regulation of lipid storage and utilization to putative involvement in obesity and metabolic diseases such as insulin resistance, type II diabetes and hepatic steatosis [40]. Studies with hepatitis C virus (HCV) have demonstrated that LDs and their close association with the ER create a unique microenvironment that promotes efficient viral replication and supports persistent viral infection [58]. The HCV Core protein has been shown to associate with LDs to form a platform for viral replication and assembly [59]. HCV Core protein expression has also been shown to modify the distribution of LDs within the cell, such that they aggregate in the perinuclear area [60], [61]. In these studies, we find that early after DENV infection, LDs are depleted and reabsorbed into the rearranged and enlarged ER. Depletion of LDs during DENV-2 infection may be utilized as a source of ready-made lipids for membrane expansion, prior to subsequent induction of pathways that respond to ER stress and lipid biosynthesis. We have previously shown the UPR and SREBP-2 pathway to be induced in a time-dependent manner during DENV-2 infection [26] consistent with studies on WNV_kun_ [35], [62]. Previous studies have also demonstrated the increase in LDs, following ER stress, to play a crucial role in sequestering misfolded proteins and preventing accumulation of lipotoxic non-esterified fatty acids by allowing their translocation to the cytosol where there are targeted to the proteasome for degradation [63]. Interestingly, the capsid protein of DENV-2 was recently shown to associate with LDs and was required for efficient viral replication [64], while a different study demonstrated that DENV-2 infection induced autophagy-dependent LD modification at later times post-infection [65]. In our study, however, the ER-reabsorbed LDs remained in their neutral state as demonstrated by the neutral lipid staining with Bodipy. Studies using Huh-7 cells have demonstrated microsomal triglyceride transfer protein (MTP) to be involved in the transmembrane transfer of neutral lipids from the cytosol to the lumen of the ER [55]. How DENV-2 infection induces the reabsorption of LDs into the ER at these early time-points, and whether it involves MTP or some other form of lipid exchange between cytosolic LDs [66] or regression [67], remains to be further investigated. Nonetheless, our studies suggest an early role for LDs in the early expansion of the ER during DENV-2 infection.

In conclusion, we have demonstrated that ER rearrangement and expansion is an early event in the DENV life cycle that is driven by viral but not host protein synthesis, is independent of the UPR and SREBP-2 pathways, and likely involves reabsportion of LDs. Our findings also demonstrate that DENV infection can be use as a tractable system for studying membrane rearrangement and LD biology. Lastly, understanding the molecular mechanism by which DENV-2 is able to hijack host cellular processes will aid in the discovery of novel anti-viral therapeutic approaches.

## Materials and Methods

### Cell Lines and Viruses

Human fibrosarcoma 2fTGH cells (kindly provided by George R. Stark, Cleveland Clinic Foundation, Cleveland, OH) [68] were grown in Dulbecco’s modified Eagle’s medium (DMEM) (Gibco BRL, Carlsbad, CA), 100 units/ml penicillin, 100 µg/ml streptomycin, and 10% fetal bovine serum (FBS) (HyClone, Logan, UT) at 37°C in 5% CO_2_. Baby hamster kidney cells (BHK-21 clone 15; kindly provided by S Kliks, San Francisco, CA) [69] were grown in minimal essential medium-alpha (MEM-α; Gibco BRL), 100 units/ml penicillin, 100 µg/ml streptomycin, and 10% FBS (HyClone). *Aedes albopictus* (C6/36) cells (American Type Culture Collection [ATCC], Manassas, VA), were grown in Leibovitz’s L-15 medium (Gibco) with 100 units/ml penicillin, 100 µg/ml streptomycin, 10 mM HEPES, and 10% FBS. XBP1^+/+^, XBP1^−/−^, ATF6α^+/+^, and ATF6α^−/−^ MEFs (kind gift from Randal Kaufman, University of Michigan, Ann Arbor, MI) were grown in DMEM, 100 units/ml penicillin, 100 µg/ml streptomycin, 1× non-essential amino acids, 55 µM β-mercaptoethanol, 1× glutamax (Gibco), and 10% FBS. DENV-2 strain PL046 (kindly provided by H.-Y Lei, National Cheng Kung University, Tainan, Taiwan) was amplified in C6/36 cells, and PFU/ml were determined using BHK cells as previously described [26].

### Chemical Treatment

2fTGH cells were mock-infected or infected with DENV-2 (PL046) at a multiplicity of infection (MOI) of 5 in the presence or absence of increasing doses of lovastatin (0–10 µM). Viral supernatants were collected at the indicated time-points, and viral titers were determined. For all confocal imaging experiments, 10 µM lovastatin was added at the time of infection. Thapsigargin (Sigma Chemical) treatment of 2fTGH cells was performed at a final concentration of 1 µM thapsigargin for 12 h. 2fTGH treatment with dithiothreitol (DTT; Sigma Chemical) was performed at a final concentration of 2 mM for the indicated times. Cycloheximide (CHX; Sigma Chemical) treatment of 2fTGH cells was performed at the time of infection at a final concentration of 50 µg/mL.

### Immunoblot Analysis

Cell lysates were prepared using RIPA lysis buffer (10 mM Tris-HCl [pH 7.5], 500 mM NaCl, 1 mM EDTA, 0.5% Na-deoxycholate, 0.1% SDS, and 1% Triton X-100) containing complete EDTA-free protease inhibitor and phosphatase inhibitors. Equal amounts of protein, as measured by Bradford assay (Bio-Rad, Hercules CA), were resolved on a 7% or 11% sodium dodecyl sulfate-polyacrylamide gel (SDS-PAGE). Proteins were transferred onto Immobilon-P polyvinylidene fluoride (PVDF) membranes (Millipore, Billerica, MA). Immunoblots were blocked at room temperature for 1 h using blocking buffer (1× phosphate-buffered saline pH 7.3 [PBS; 137 mM NaCl, 2.7 mM KCl, 4.3 mM Na_2_HPO_4_•7H_2_O, 1.4 mM KH_2_PO_4_], 5% non-fat dry milk, and 0.1% Tween-20).

Primary antibodies were obtained from the following sources and were diluted in blocking buffer at the following dilutions: anti-GRP78 (1∶1,000), anti-actin-horseradish peroxidase (HRP) (1∶1,000) (Santa Cruz Biotechnologies); anti-SREBP-2 (1∶1,000) (BD Pharmigen); anti-HMG-CoA reductase (1∶1,000) (Upstate Biotechnologies); and anti-NS1 mouse monoclonal antibody (MAb) 7E11 (1∶1,000), which recognizes all four DENV serotypes (kind gift from Robert J. Putnak, Walter Reed Army Institute of Research, Washington, DC). The blots were incubated overnight, washed twice with 1× PBS-T (PBS containing 0.1% Tween-20) at room temperature, and incubated for 1 h with HRP-conjugated goat anti-rabbit or goat anti-mouse at 1∶10,000 or 1∶5,000, respectively. Images were aquired using the Chemi-Doc system (Bio-Rad).

### Confocal Microscopy

2fTGH cells and ATF6^+/+^, ATF6^−/−^, XBP1^+/+^ and XBP1^−/−^ MEF cells were seeded at 5×10^4^ cells/well in a 12-well plate containing a microscope cover slip in each well and left to adhere overnight. Cells were infected with DENV-2 at a MOI of 5 for 12 h. Cover slips were washed three times with 1× PBS, fixed in 4% formaldehyde for 10 minutes, and washed three times with 1× PBS. Cells were then permeabilized using 0.5% saponin in PBS for 10 minutes. Blocking was performed using 1% BSA in 0.1% Tween-PBS buffer for 1 h. Primary antibodies were diluted 1∶50 in blocking buffer for 1 h and were obtained from the following sources: rabbit anti-GRP78, rabbit anti-GRP94, goat anti-ATF6 (N16) and rabbit anti-XBP1 (Santa Cruz Biotechnologies); mouse anti-SREBP-2 (BD Pharmigen); and rabbit anti-HMG-CoA reductase (Upstate Biotechnologies). Mouse anti-DENV E (4G2; ATCC) and mouse anti-NS3 (E1D8; P.R. Beatty and E. Harris, unpublished) antibodies were directly conjugated to Alexa-488 and Alexa-594, respectively. Alexa Fluor™ 488 (goat anti-rabbit), Alexa Fluor™ 546 (donkey anti-goat), and Alexa Fluor™ 647 (goat anti-rabbit) were diluted 1∶200 (Molecular Probes, Invitrogen Corporation, Carlsbad, CA). Cover slips were mounted using VECTASHIELD® mounting medium with DAPI (Vector Laboratories Burlingame, CA). Images were captured using the 63×/1.25 Plan-Neofluar DIC oil objective on a Zeiss laser-scanning confocal microscope (LSM) 510 UV/Vis or using a 40×/1.4 Plan-Apochromat DIC oil objective on a Zeiss LSM 710 Meta LSM (College of Natural Resources Biological Imaging Facility, UC Berkeley, Berkeley, CA). All images were processed using Photoshop CS4 Suites and Zeiss operating software.

To generate the 3D image in Fig. 7B, 2fTGH cells were infected with DENV-2 and fixed and permeabilized as described above. Cells were then incubated with mouse MAb against DENV-2 E-protein overnight. Cells were washed twice with PBS-Tween and stained with goat anti-mouse secondary antibody conjugated to Alexa Fluor™647 for 1 h. Cells were then washed again and incubated with mouse anti-NS3 MAb directly conjugated to Alexa-594 for 1 h, at which time Bodipy 493/503 (Molecular Probes) was added at a final concentration of 20 µg/mL and incubated for an additional 15 minutes. Cells were washed and mounted as described above. Optical slices were acquired at 0.1 µM Z-stacking intervals. These stacks were then deconvoluted using Huygens Essential Software (Scientific Volume Imaging), and a three-dimensional image was generated using Imaris 7.2 software (Bitplane Scientific Software).

## Supporting Information

Figure S1 **Controls for UPR markers in 2fTGH cells.** 2fTGH cells were (A) mock-treated or (B) treated with DTT for 12 h, fixed and stained intracellularly for cellular proteins XBP1 (green) and ATF6 (red), followed by secondary antibodies conjugated to Alexa Fluor® 488 and Alexa Fluor® 594, respectively. Mock-infected 2fTGH cells were also treated with 1 µM thapsigargin for 12 h, then stained intracellularly for cellular proteins (C) GRP78 (magenta) and (D) GRP94 (magenta), followed by secondary antibodies conjugated to Alexa Fluor® 647 and mouse monoclonal antibodies directly conjugated to viral proteins E (green) and NS3 (red). GRP78 (E) and GRP94 (F) cellular expression in mock-infected and mock-treated 2fTGH cells. Images were obtained as previously described in Materials and Methods. Total magnification 400×.
(TIF)

Figure S2 **Lovastatin inhibits infectious DENV viral production.** 2fTGH cells were infected with DENV-2 and treated with increasing concentrations of lovastatin (0–10 µM) at the time of infection. Supernatants were collected at the indicated time-points, and infectious virus production was determined by plaque assay. Error bars represent +/− SD; n = 3.
(TIF)

Figure S3 **Actinomycin D inhibition of thapsigargin-induced GRP78 expression in 2fTGH cells.** 2fTGH cells were mock-treated or treated with 1 µM thapsigargin (Tg) or 50 µg/mL actinomycin D (Act D) or both for 12 h, and levels of GRP78 were analyzed by immunoblot analysis as previously described.
(TIF)
