# Supplementary figures and images for: Early Dengue Virus Protein Synthesis Induces Extensive Rearrangement of the Endoplasmic Reticulum Independent of the UPR and SREBP-2 Pathway
Source: PLoS One. 2012 Jun 4;7(6):e38202. doi: 10.1371/journal.pone.0038202 (PMC3366941; doi:10.1371/journal.pone.0038202)

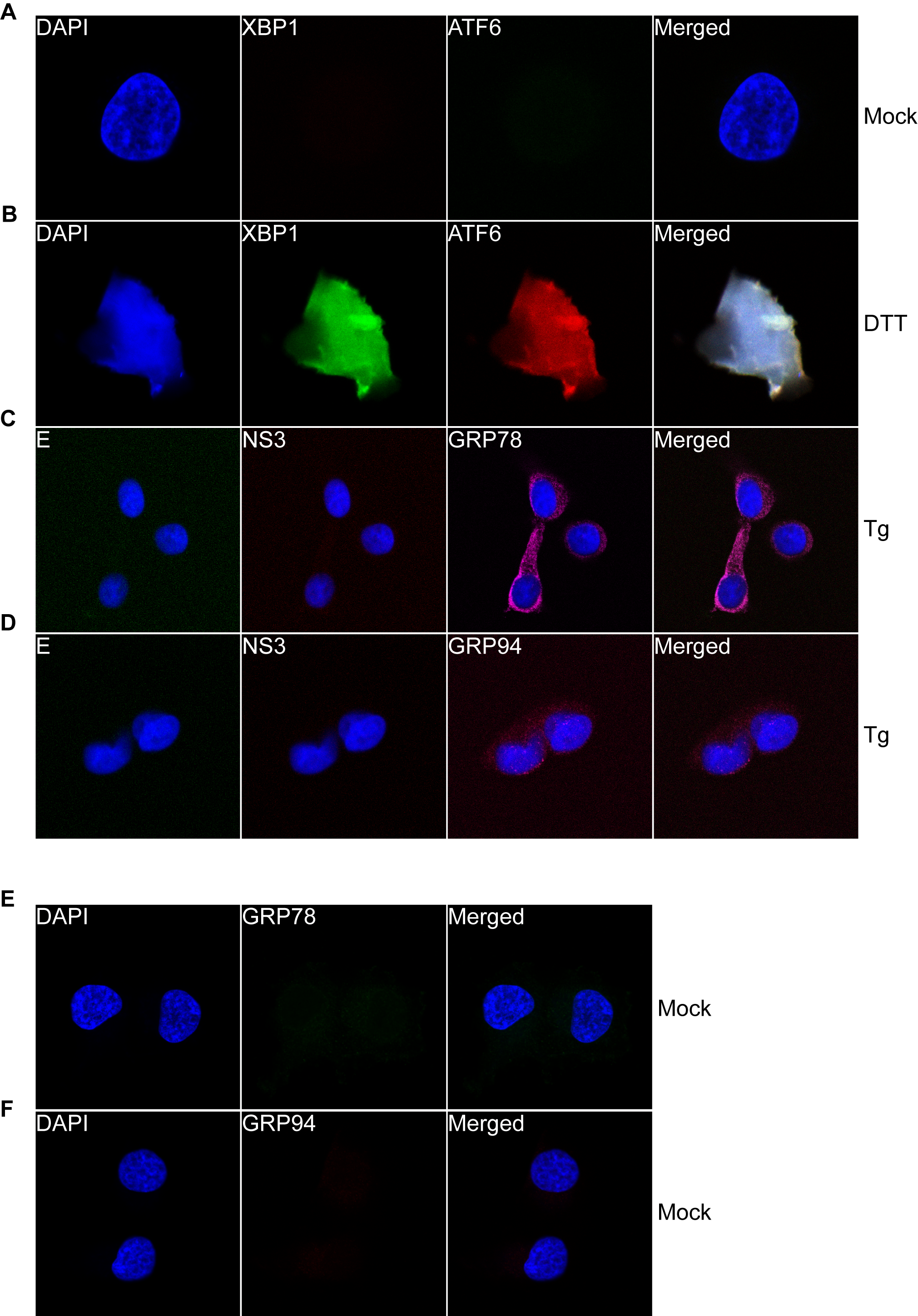

Supplement: Figure S1 — Controls for UPR markers in 2fTGH cells. 2fTGH cells were (A) mock-treated or (B) treated with DTT for 12 h, fixed and stained intracellularly for cellular proteins XBP1 (green) and ATF6 (red), followed by secondary antibodies conjugated to Alexa Fluor® 488 and Alexa Fluor® 594, respectively. Mock-infected 2fTGH cells were also treated with 1 µM thapsigargin for 12 h, then stained intracellularly for cellular proteins (C) GRP78 (magenta) and (D) GRP94 (magenta), followed by secondary antibodies conjugated to Alexa Fluor® 647 and mouse monoclonal antibodies directly conjugated to viral proteins E (green) and NS3 (red). GRP78 (E) and GRP94 (F) cellular expression in mock-infected and mock-treated 2fTGH cells. Images were obtained as previously described in Materials and Methods. Total magnification 400×. (TIF) [file pone.0038202.s001.tif]

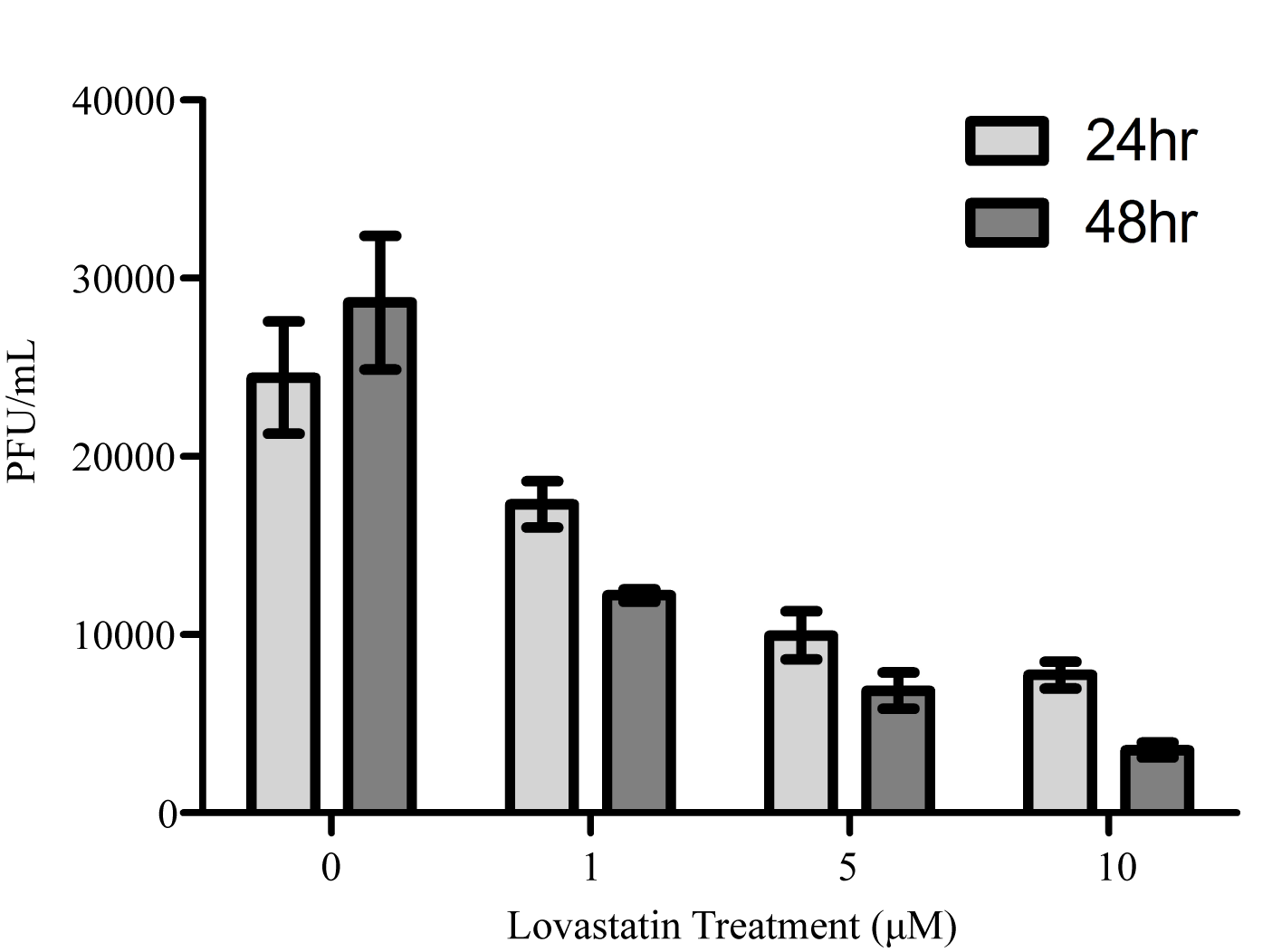

Supplement: Figure S2 — Lovastatin inhibits infectious DENV viral production. 2fTGH cells were infected with DENV-2 and treated with increasing concentrations of lovastatin (0–10 µM) at the time of infection. Supernatants were collected at the indicated time-points, and infectious virus production was determined by plaque assay. Error bars represent +/− SD; n = 3. (TIF) [file pone.0038202.s002.tif]

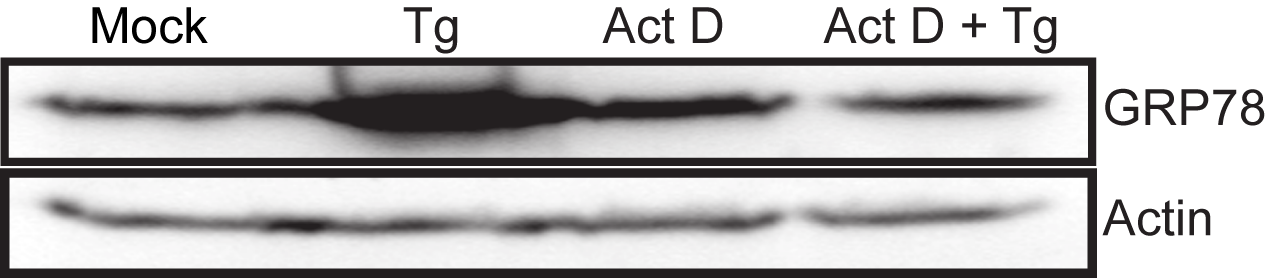

Supplement: Figure S3 — Actinomycin D inhibition of thapsigargin-induced GRP78 expression in 2fTGH cells. 2fTGH cells were mock-treated or treated with 1 µM thapsigargin (Tg) or 50 µg/mL actinomycin D (Act D) or both for 12 h, and levels of GRP78 were analyzed by immunoblot analysis as previously described. (TIF) [file pone.0038202.s003.tif]
